# High PEEP may have reduced injurious transpulmonary pressure swings in the ROSE trial

**DOI:** 10.1186/s13054-019-2689-x

**Published:** 2019-12-11

**Authors:** João B. Borges, Caio C. A. Morais, Eduardo L. V. Costa

**Affiliations:** 1 Centre for Human & Applied Physiological Sciences (CHAPS), King’s College London, London, UK; 2 Divisao de Pneumologia, Instituto do Coracao, Hospital das Clinicas HCFMUSP, Faculdade de Medicina, Universidade de Sao Paulo, Sao Paulo, Brazil

Vigorous spontaneous inspiratory efforts can lower pleural pressures and increase transpulmonary pressures, worsening existing lung injury. Muscle paralysis [1] may prevent breath stacking and pendelluft associated with high respiratory drive and very negative pleural pressures. A recent and comprehensive study [2] compared two positive end-expiratory pressure (PEEP) strategies and found that an oxygenation-based method to select PEEP resulted in strong inspiratory efforts, high local lung stress, and intensely focused inflammation in dependent lung regions. In contrast, high PEEP rendered spontaneous effort less injurious by lowering the level of spontaneous effort via neuromechanical uncoupling (Fig. 1) and by converting solid-like (more atelectatic) lung to fluid-like (less atelectatic) lung, reducing the vertical gradient of inspiratory local negative swings in pleural pressure. Both mechanisms worked together to promote a more homogeneous lung expansion. One of the limitations of a high PEEP strategy is that mechanical ventilation with PEEP may result in longitudinal atrophy of diaphragm fibers [3]. We postulate that in the ROSE trial [4, 5] the use of lung-protective ventilation with high PEEP (instead of a ventilation strategy with low PEEP [6]) reduced potentially injurious transpulmonary pressure swings in both groups, making the muscle paralysis unnecessary and preventing the potential harmful effects of strong spontaneous efforts in moderate-to-severe ARDS.

**Fig. 1:**
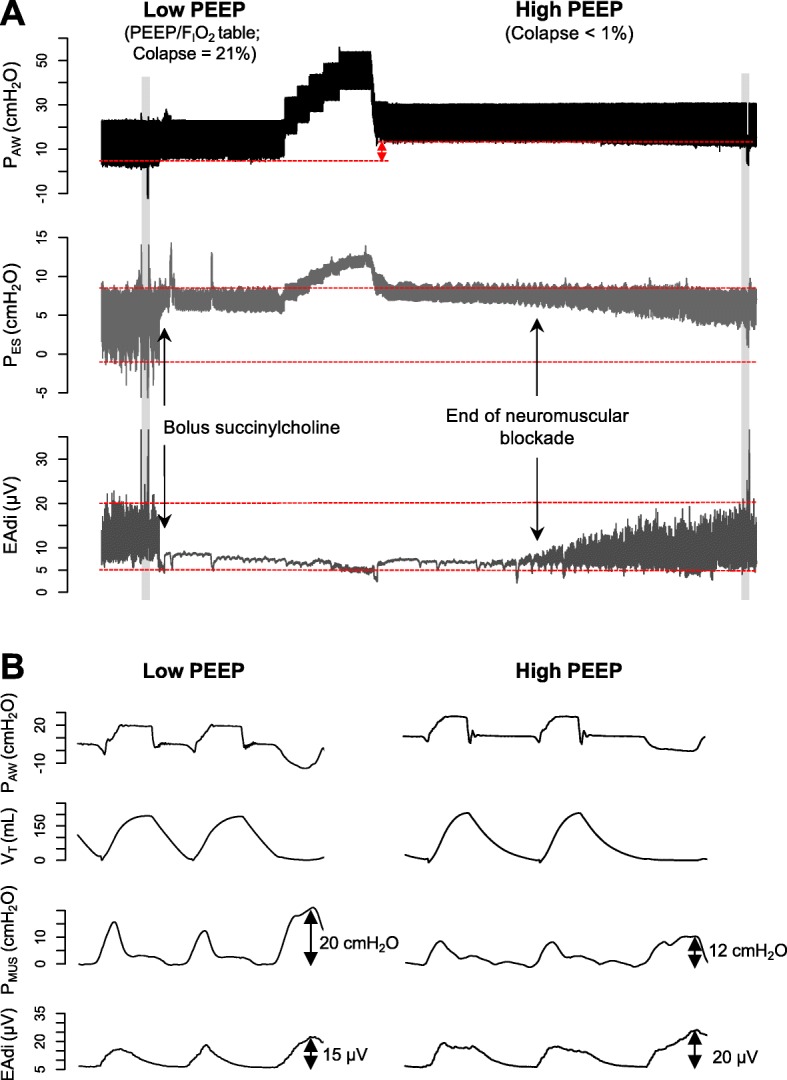
Intensity of spontaneous effort with low vs. high positive end-expiratory pressure (PEEP) in an experimental model of severe acute respiratory distress syndrome. **a** Representative waveforms of airway pressure (*P*_AW_), esophageal pressure (*P*_ES_), and electrical activity of the diaphragm (EAdi). Note that the magnitude of the negative swings of esophageal pressure was reduced by approximately 50% when PEEP was increased from 7 cmH_2_O (low PEEP/F_I_O_2_ table, corresponding to lung collapse = 21%) to 15 cmH_2_O [PEEP level individually titrated by electrical impedance tomography (EIT), corresponding to lung collapse < 1%], with similar EAdi. **b** A zoom into the shaded areas that highlight the induced neuromechanical uncoupling when PEEP was increased, that is, less pressure generated by the respiratory muscles (*P*_MUS_) for each microvolt of electrical activity (*P*_MUS_/EAdi index during low and high PEEP = 1.33 cmH_2_O/μV vs. 0.6 cmH_2_O/μV, respectively)

## Data Availability

The datasets used and/or analyzed reported here are available from the corresponding author on reasonable request.
